# Extracellular Vesicles Work as a Functional Inflammatory Mediator Between Vascular Endothelial Cells and Immune Cells

**DOI:** 10.3389/fimmu.2018.01789

**Published:** 2018-08-06

**Authors:** Baharak Hosseinkhani, Sören Kuypers, Nynke M. S. van den Akker, Daniel G. M. Molin, Luc Michiels

**Affiliations:** ^1^Department of Medicine and Life Sciences, Biomedical Research Institute (BIOMED), Hasselt University, Hasselt, Belgium; ^2^Department of Physiology, Cardiovascular Research Institute Maastricht (CARIM), Maastricht University, Maastricht, Netherlands

**Keywords:** extracellular vesicles, cardiovascular disease, inflammation, monocyte, endothelial cells

## Abstract

Extracellular vesicles (EV) mediated intercellular communication between monocytes and endothelial cells (EC) might play a major role in vascular inflammation and atherosclerotic plaque formation during cardiovascular diseases (CVD). While critical involvement of small (exosomes) and large EV (microvesicles) in CVD has recently been appreciated, the pro- and/or anti-inflammatory impact of a bulk EV (exosomes + microvesicles) on vascular cell function as well as their inflammatory capacity are poorly defined. This study aims to unravel the immunomodulatory content of EV bulk derived from control (uEV) and TNF-α induced inflamed endothelial cells (tEV) and to define their capacity to affect the inflammatory status of recipients monocytes (THP-1) and endothelial cells (HUVEC) *in vitro*. Here, we show that EV derived from inflamed vascular EC were readily taken up by THP-1 and HUVEC. Human inflammation antibody array together with ELISA revealed that tEV contain a pro-inflammatory profile with chemotactic mediators, including intercellular adhesion molecule (ICAM)-1, CCL-2, IL-6, IL-8, CXCL-10, CCL-5, and TNF-α as compared to uEV. In addition, EV may mediate a selective transfer of functional inflammatory mediators to their target cells and modulate them toward either pro-inflammatory (HUVEC) or anti/pro-inflammatory (THP-1) mode. Accordingly, the expression of pro-inflammatory markers (IL-6, IL-8, and ICAM-1) in tEV-treated HUVEC was increased. In the case of THP-1, EC-EV do induce a mixed of pro- and anti-inflammatory response as indicated by the elevated expression of ICAM-1, CCL-4, CCL-5, and CXCL-10 proteins. At the functional level, EC-EV mediated inflammation and promoted the adhesion and migration of THP-1. Taken together, our findings proved that the EV released from inflamed EC were enriched with a cocktail of inflammatory markers, chemokines, and cytokines which are able to establish a targeted cross-talk between EC and monocytes and reprogramming them toward a pro- or anti-inflammatory phenotypes.

**Graphical Abstract:**
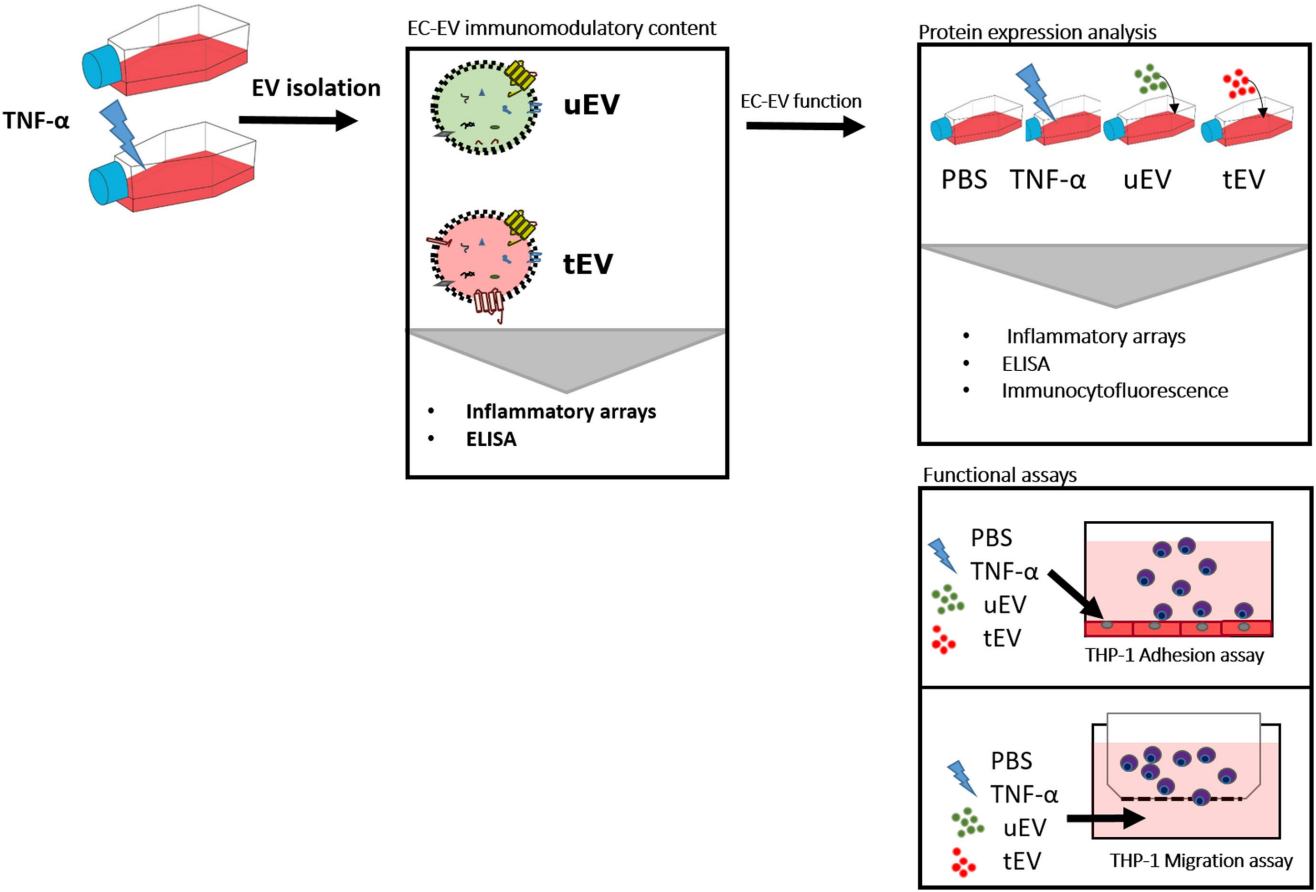
Immunomodulatory content of bulk extracellular vesicles (EV) (exosomes + microvesicles) derived from untreated (uEV) and TNF-α treated endothelial cells (tEV) and unraveling the functional inflammatory impact of uEV and tEV on the phenotype and behavior of monocytes (THP-1) and endothelial cells (HUVEC) *in vitro*.

## Introduction

Atherosclerosis is a chronic and progressive inflammatory vascular disorder that largely contributes to the development of cardiovascular diseases (CVD) including coronary artery and peripheral vascular disease (1). Tightly regulated inflammatory interactions between two major cellular players, monocytes (MC) and endothelial cells (EC), play a pivotal role in atherosclerotic plaque formation in the arterial intima (2). EC have been known as the major functional coordinator in the cardiovascular homeostasis and maintaining cardiac functions (3). Accumulating epidemiological and clinical evidence in CVD since 1970 suggest that traditional risk factors such as smoking, elevated blood sugar, hypertension, diabetes, infection, homocysteine, ischemia, and oxidant damage evoke endothelial dysfunction and reprogram them toward either pro- and/or anti-inflammatory actions (4). Accordingly, overexpression of adhesion molecules [e.g., vascular cell adhesion molecule-1, intercellular adhesion molecule (ICAM)-1] on the EC surface together with the secretion of cytokines and chemokines lead to the recruitment of circulating MC into the intima (5, 6). Functionally, transmigrated MC will initiate the formation of atherosclerotic plaques, termed fatty streaks, in the arterial walls that, in turn, will lead to CVD (7). So far, the communication of EC with their neighboring EC as well as circulating MC during development of CVD is largely unknown. In recent times, the findings of extracellular vesicles (EV) have opened new perspectives in the understanding of cell–cell communication during the development of several diseases including CVD (8). EV, traditionally classified as exosomes (40–100 nm), microvesicles (100 nm–1 μm), and apoptotic bodies (>1 μm), have received extensive attention as a novel cell free-signaling conveyors of bioactive molecules in the body fluids and, which can have dramatic impact on the fitness of their recipient cells (9, 10). However, many studies have been focusing on the participation of a certain fraction of EV (e.g., exosome) in the progression of CVD at RNA level (11, 12). In spite of that, the protein profile of EV and their mode of action at the site of inflamed vascular cells are still not well defined. In this study, we first aim to unravel the immunomodulatory content of EV bulk derived from inflammatory-triggered EC, thereafter, to understand their pathological and functional impact on the cellular profiles and behavior of recipient cells.

In order to understand the underlying mechanism of the involvement of EV in the cross-talk between two CVD key players (EC and MC), transmission electron microscopy (TEM), nanosight tracking analysis (NTA), and western blot were used to confirm the presence of EV (exosomes + microvesicles) in the culture supernatant of a human vascular endothelial cell model (HUVEC), either untreated (uEV) or treated with TNF-α to induce an inflammatory stress (tEV). Furthermore, human inflammation antibody arrays were used to discover the immunomodulatory content of both uEV and tEV. Thereafter, HUVEC and a circulating human MC model (THP-1) were exposed to uEV or tEV. Relevant pro/anti-inflammatory markers [IL-1β, IL-4, IL-6, IL6-R, IL-8 (CXCL8), IL-10, IL-13, TNF-α, ICAM-1, CCL-2 (MCP-1), CD-40, HSP-70, CXCL-10 (IP-10), CCL-4 (MIP-1), CCL-5 (RANTES), TIMP-2] were evaluated at the protein in both cell types. Furthermore, the functional inflammatory effect of uEV and tEV was assessed using *in vitro* monocyte adhesion and migration assays. We discovered that EV may selectively transfer functional inflammatory mediators to their target cells. Accordingly, they were dramatically altering the cellular profile of their recipients toward either pro-inflammatory (HUVEC) or anti/pro-inflammatory (THP-1) *via* the expression of several inflammatory markers. In addition, these biologically active EV induced the THP-1 migration and the adhesion of THP-1 into HUVEC. Altogether, our current findings for the first time highlighted that the EV released from inflamed EC were enriched with a cocktail of inflammatory proteins, chemokines, and cytokines. These findings also demonstrate that EC-EV are able to establish a targeted cross-talk between EC and MC as well as reprogramming them toward a pro- or anti-inflammatory phenotypes, resulting in the adhesion and mobilization of MC.

## Materials and Methods

### Reagents

The following primary antibodies were applied in this study: mouse monoclonal anti-human intercellular adhesion molecule-1 (clone 15.2, Santa Cruz, sc-107), CD63 (clone Ts63, Thermo Fisher) and CD9 (clone Ts9, Life Technologies), GM-130 (610822, BD Biosciences), β-actin (Santa Cruz), Rabbit anti-mouse HRP-conjugated secondary antibody (Dako, P0260) and donkey anti-mouse IgG, Alexa Fluor^®^ 488 antibody (clone A-21202, Thermos Fisher). Calcein, AM (C3099a), CellMask™ orange plasma membrane stains (CS10045), and Hoechst 33342 were obtained from Thermo Fisher Scientific. 4, 6 diamidino-2-phenylindole (DAPI) was provided by Sigma-Aldrich.

### Cells and Culture Conditions

HUVEC (BD Bioscience, cat # 354151) at passages three to six were seeded at a density of 600,000 cells in EBM-2 (Lonza) supplemented with EGM-2 MV SingleQuot Kit (Lonza) and 5% vesicles-depleted fetal bovine serum (System Bioscience). When HUVEC were grown up to 70–75% confluency, cells were washed twice with HEPES buffer saline (Lonza) and cells were then inflammatory triggered by adding 10 ng/ml TNF-α in refreshed medium for overnight (13). Afterward, the supernatants were collected for the EV isolation. All collected supernatant samples containing EV were stored at −80°C until EV isolation procedures.

THP-1 (ATCC^®^ TIB-202™) were grown in RPMI-1640 (Life Technologies) medium supplemented with 10% vesicles-depleted fetal bovine serum (System Bioscience) and 1% penicillin–streptomycin–amphotericin B (Lonza Biowhittaker). All cell lines were incubated in a humidified atmosphere condition of 5% CO_2_/95% O_2_ at 37°C.

### EV Isolation

A modified differential centrifugation method was used to collect the bulk EC-EV containing large EV (microversicle) and small EV (exosomes) from cell culture supernatant of unstimulated (uEV), TNF-α stimulated (tEV), and cell-free medium (cEV). Briefly, collected supernatant from the same number of parent cells was first centrifuged at 300 *g* for 5 min at 4°C to eliminate cell debris. To remove remaining debris and apoptotic bodies, another centrifugation step was done on the supernatant passed through a 0.22-µm filter (VWR, Belgium) for 20 min at 2,000 *g* at 4°C (14). Afterward, to pellet the EC-EV, the supernatant was centrifuged at 110,000 *g* for 3 h at 4°C. All ultracentrifugation (UC) steps were performed using an L-90 Beckman centrifuge (Beckman Instruments, Inc., Fullerton, CA, USA) equipped with a Ti-70 rotor (Beckman Instruments) (15). Based on the downstream analysis, pellets were suspended in 1 ml of HEPES (Lonza), RIPA or extraction buffers (Abcam).

### Nanosight Tracking Analysis

Extracellular vesicles size distribution and concentration were analyzed based on the tracking of light scattered by vesicles moving under Brownian motion using the NanoSight NS300 system (Sysmex Belgium N.V.) equipped with a 532-nm laser. The data were captured and analyzed using NTA software 3.2 (NanoSight Ltd.). Samples were diluted with PBS over a range of concentrations to obtain between 20 and 50 particles per frame. Samples were injected into the sample chamber and measured three times for 60 s at 25°C with manual shutter and gain adjustments for three individual samples.

### Transmission Electron Microscopy

Transmission electron microscopy samples were prepared and analyzed as previously described (16). The size and morphology of EC-EV were evaluated using a Tecnai G2 transmission electron microscope (TEM; Tecnai G^2^ spirit twin, FEI, Eindhoven, the Netherlands) at 120 kV. The microscope was provided with a bottom mounted digital camera FEI Eagle (4k × 4k pixels) to acquire images of the evaluated samples. Digital processing of the images was performed with the FEI imaging software (TEM Imaging and Analysis version 3.2 SP4 build 419).

### Live Imaging

Labeling of EC-EV and cEV was performed by adding 50 µg/ml CellMask™ orange plasma membrane tracking label for 10 min at 37°C into the supernatant. Free dye was removed from labeled EV using Amicon^®^Ultra centrifugal columns (10 kDa cutoff) after isolation procedures. Labeled EVs were added to approximately 1 × 10^6^ of HUVECs cell per well in an eight-well culture plate (Ibidi GmbH, Martinsried, Germany). In the case of THP-1, labeled EV were added into poly-d-lysine-coated glass coverslips (Sigma) which were seeded overnight with 8 × 10^5^ undifferentiated THP-1 in six-well plates. Following 2–24 h of incubation, the live cell imagining of internalized of EV was performed using Zeiss LSM 510 META confocal laser scanning microscope (Jena, Germany) on an Axiovert 200 M motorized frame for TICS, STICS, and STICCS analyses. The microscope was coupled to a 30 mW air-cooled argon ion laser emitting at 488 nm under the control of an acousto-optic modulator (~11 µW irradiance at the sample position) for one-photon excitation. To provide a suitable environment for sustaining cells during the imaging steps, the microscope was equipped with an airtight chamber (Tempcontrol 37–2 digital, PeCon, Erbach, Germany) with controlled temperature at 37°C. Cell-free medium-derived EV served as a negative control. Nuclei were stained with Hoechst 33342.

### Protein Quantification

Extracellular vesicles protein lysates in RIPA buffer for western blotting, EV protein lysates in extraction buffer (ab193970, Abcam Ltd., Cambridge, UK) for ELISA and inflammatory cytokine arrays and EV suspensions for migration and adhesion assays were quantified using the Pierce BCA Protein Assay Reagent Kit (Thermo Scientific Pierce, USA) following the manufacturer’s protocol. Optical density of standards and samples were measured at OD595 nm using a Multiskan™ FC Microplate Absorbance Reader (Thermo Scientific, Belgium).

### Inflammatory Cytokine Arrays

To simultaneously detecting and semi-quantifying of 40 inflammatory markers in EV and cell lysates, human cytokine antibody C1, C2, and C3 arrays were purchased from RayBiotech (Boechout, Belgium). Experiments were done according to the manufacturers’ instructions. Briefly, 25 µg of EV lysate or cell lysate proteins in extraction buffer (ab193970, Abcam Ltd., Cambridge, UK) were added in to a pre-blocked membrane and incubated overnight at 4°C with gentle shaking. Thereafter, the membrane incubated with the primary biotin-conjugated antibody for 2 h, followed by incubation with HRP-conjugated streptavidin antibodies for 1 h. The signal intensity of each array was scanned by densitometry using the ImagerQuant™TL detection system. Intensity of each dots was then quantified using ImageJ open source software (National Institutes of Health, USA). Heat maps of inflammation-related protein expression was analyzed using GENE-E open source software.

### ELISA

Quantification of several inflammatory cytokines [IL1-β (ab46052), IL-4 (ab100570), IL-6 (ab46027), IL-6R (ab46029), IL-8 (ab46032), IL-10 (ab46034), IL-13 (ab100553)], cell adhesion markers [ICAM-1 (ab174445), CCL-2 (MCP-1, ab179886)], chemokines [CCL-4 (MIP-1β, ab100597), CCL-5 (RANTES, ab174446), CXCL-10 (IP-10, ab83700), TIMP-2 (ab100653)], and other known CVD marker [CD-40 (ab99990) and HSP-70 (ab187399)] were performed and normalized for 1 µg total protein of cell lysates and EV lysates using Human ELISA Kits (Abcam Ltd., Cambridge, UK), according to their manufacturer’s instructions. Cell-free medium-derived EV (cEV) served as a negative control. Optical density of standards and samples were measured using a Multiskan™ FC Microplate Absorbance Reader (Thermo Scientific, Belgium).

### Western Blotting

The equivalent of 5 µg of EV proteins in RIPA buffer containing protease inhibitor cocktail (Sigma-Aldrich) were first separated by SDS-PAGE with 8 or 12% polyacrylamide gels under 200 V for 30–45 min. The proteins were then electrophoretically transferred to a polyvinylidene fluoride membrane for minimum 1 h at 350 mA. The membranes were blocked with PBS Marvel 5% for 2 h and incubated with 1:1,000 dilution of primary antibodies against CD9, CD63, ICAM-1, GM-130 (negative control), and β-actin (reference protein) overnight at 4°C. Next, rabbit anti-mouse HRP-conjugated secondary antibody at 1:2,000 dilution (Agilent, USA) were added in to the membrane for 1 h at room temperature (RT). The blots were developed with Pierce™ ECL Western Blotting Substrate. The corresponding bands were detected by the ImagerQuant™TL detection system. Intensity of each bands (2×) was quantified using ImageJ open source software (National Institutes of Health, USA) (17).

### Immunofluorescence Staining

HUVECs were first grown into four-well culture slides (Sarstedt, Berchem, Belgium) up to 70–75% confluency. Cells were then stimulated with PBS, 10 ng/ml TNF-α (ImmunoTools), uEVs or tEVs for 24 h. An equal amount of EV with total protein concentration (10 µg/ml) was added to the cell cultures with the use of the BCA-assay results. After treatment, HUVEC were fixated and permeabalized with 4% paraformaldehyde for 10 min at RT and then rinsed with PBS twice. Specimens were incubated with the corresponding primary antibody against ICAM-1 (1:500 in PBS) for overnight at RT. After three times washing with PBS (Lonza), the secondary antibody donkey-anti-mouse Alexa 488 (1:1,000 in PBS, Thermo Fisher Scientific) was applied into each chamber for 1 h at RT in the dark. Nuclei were stained with DAPI. Images were taken with a Leica DM4000 B LED microscope along with a digital microscope camera Leica DFC450 C (Leica, Diegem, Belgium). ImageJ open source software (National Institutes of Health, USA) was used to calculate the mean of fluorescence intensity (MFI) for each protein of interest under different treatments in HUVEC and THP-1. The MFI was measured by subtracting the multiplication of the area of the selected cell and the mean fluorescence of the background readings from the integrated density of each cell.

### Transmembrane Migration Assay

THP-1 cells were harvested from RPMI-1640 medium supplemented with 10% FBS and washed twice with PBS, then, incubated in serum free medium for 2 h. EV samples in the experiments were diluted in RPMI-1640 medium containing 0% FBS. The migration capacity of THP-1 was determined using 8 µm pore polycarbonate filter transwell plates (ThinCert Cell Culture Inserts, Greiner bio-one, Vilvoorde, Belgium). Briefly, 300 µl of the above prepared THP-1 (10^6^ cells/ml) were seeded on top of the transwell insert and the lower chambers were filled with 500 µl RPMI-1640 medium containing 0% FBS with or without 10 µg/ml of uEV and tEV samples. RPMI-1640 supplemented with 10% FBS (Thermo Fisher Scientific) and 50 ng/ml recombinant human MCP-1 (PeproTECH, Rocky Hill, CT, USA) were used as positive controls. After overnight incubation (~16 h) at 37°C, the number of cells that passed through the membrane were counted in the lower chambers using trypan blue 0.4% (Thermo Fisher Scientific). The percentage of migrated cells for each condition in three independent experiments with three technical replicates (*n* = 9) were calculated.

### Cell Adhesion Assay

HUVEC were first grown into four-well microscope slides (Sarstedt—Germany) up to 70–75% confluency. HUVEC were treated with PBS, 10 ng/ml TNF-α (ImmunoTools) and a 10 µg/ml uEVs and tEVs overnight (~16 h). Nuclei of HUVEC were stained with Hoechst33342 staining solution (Thermo Fisher Scientific) and cells washed twice with PBS to remove the non-engulfed EV and dye residuals. THP-1 cells were also grown in RPMI-1640 medium supplemented with 10% FBS. THP-1 were stained with 5 µM Calcien AM, for 15 min at 37°C, washed twice with PBS. Fluorescently labeled THP-1 were co-incubated with the pretreated HUVEC for 60 min at 37°C. Afterward, HUVEC were thoroughly washed with PBS (6×) to remove the non-adherent THP-1 cells. Images were taken with a Leica DM4000 B LED microscope supplemented with a digital microscope camera Leica DFC450 C (Leica, Belgium). ImageJ open source software (National Institutes of Health, USA) was used to calculate the percentage of adhered THP-1 monocytes to HUVEC under different treatments (18).

### Statistical Analysis

Data were presented as mean ± SD of three independent experiments in two technical replicates (*n* = 6) or three technical replicates (*n* = 9). One-way analysis of variance (ANOVA) with a multiple comparisons test (Tukey’s multiple comparison test) and Student’s test using the statistical packages GraphPad Prism 7.04 software (GraphPad Software, Inc., USA) were applied to evaluate the statistical significance between different treatments. Two-tailed tests at value of *p < 0.05 and were considered as statistically significant. NS represented as not significant, *p* > 0.05.

## Results

### Cross Internalization of EC-EV Into Vascular EC (HUVEC) and Circulating Immune Cells (THP-1)

**Figure 1:**
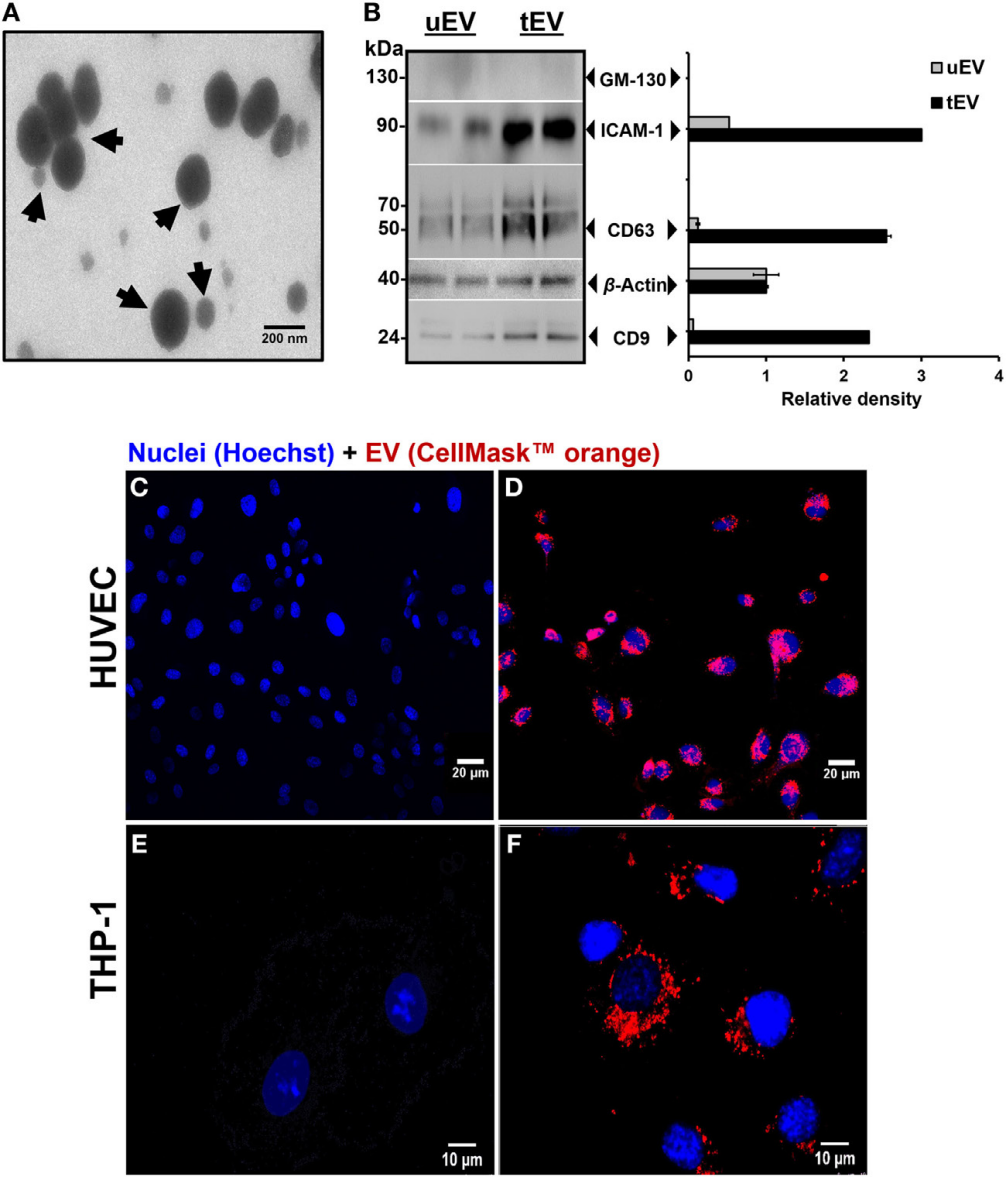
Characterization and *in vitro* cellular uptake of endothelial cells (EC)-extracellular vesicles (EV). **(A)** Transmission electron microscopy image of ultracentrifugation-purified of EC-EV bulk (black arrowheads point toward the large and small EV). Scale bar, 200 nm. **(B)** Representative western blots and densitometric analysis of CD9 (24 kDa), CD63 (30–70 kDa) as classical EV membrane-bound markers, intercellular adhesion molecule (ICAM)-1 (90 kDa) as inflammatory-associated marker, and GM-130 (130 kDa) as a Golgi marker in uEV (2×) and tEV (2×). Five micrograms of EV proteins were loaded on the gels. CD9, CD63, and ICAM-1 markers were highly enriched in tEV in comparison with uEV. The absence of GM130 in uEV and tEV confirmed the purity of samples. **(C–F)** *In vitro* internalization of fluorescently labeled EV with CellMask™ orange plasma membrane into HUVEC **(D)** and THP-1 **(F)** within 3 h. **(C,E)** No vesicles were detected in the controls. The cell nucleus was stained with Hoechst. Scale bar, 20 µm.

Extracellular vesicles bulk were pelleted from HUVEC cell culture supernatant using a modified differential UC. UC-purified EV contained a mixture of large (microvesicles) and small EV (exosomes) (TEM image: Figure 1A and NTA analysis: Figure S1 in Supplementary Material). In line with previous data, UC-isolated EV from either untreated EC (uEV) or EC treated with TNF-α (tEV) were enriched with both classical EV membrane-bound biomarkers including CD9, CD63, CD81, and ICAM-1 (16). Comparative marker analysis of selected classical (CD9 and CD63) and inflammatory (ICAM-1) associated markers was performed on the bulk of uEV and tEV using western blot. CD9 (24 kDa), CD63 (30–70 kDa), and ICAM-1 (90 kDa) were highly enriched in EV bulk derived from TNF-α stimulated HUVEC (tEV) in comparison with EV derived from unstimulated cells (uEV) (Figure 1B). GM-130 (a Golgi-related protein) was used as a negative marker protein for EV. The absence of the GM130 (130 kDa) in uEV and tEV confirmed the purity of samples. Within 3 h EV derived from EC (HUVEC) were taken up by HUVEC (Figure 1D) and THP-1 (Figure 1F) from EV-supplemented culture medium and predominantly accumulated in the perinuclear region. No vesicles were detected in the control experiments (EV isolated from cell-free medium) (Figures 1C,E). Altogether these observations confirmed that inflammatory-triggered EC secreted a bulk of EV containing large and small-sized vesicles which were taken up by vascular EC (HUVEC) and circulating immune cells (THP-1).

### EC-EV Immunomodulatory Content and Their Mode of Action

**Figure 2:**
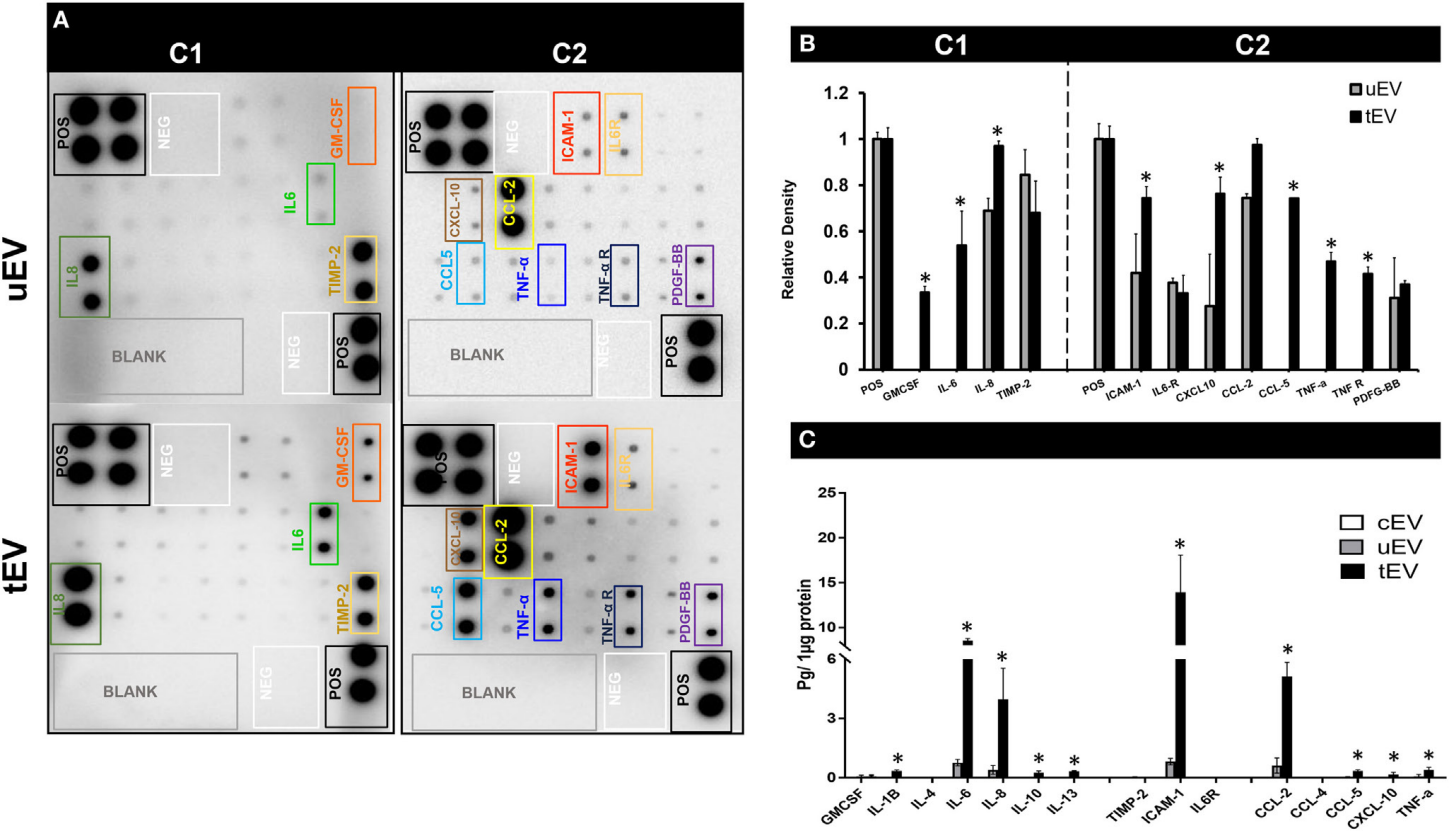
The immunomodulatory content of extracellular vesicles (EV) derived from TNF-α stimulated HUVEC (tEV) and non-stressed (unstimulated) cells (uEV). **(A)** A representative image of membrane based inflammation arrays C1 and C2 of uEV and tEV. **(B)** Relative densitometry of each protein was obtained using Image J software. *p Values <0.05 was considered as statistically significant. **(C)** ELISA analysis of GM-CSF, IL1-β, IL-4, IL-6, IL-6R, IL-8, IL-10, IL-13, intercellular adhesion molecule (ICAM)-1, CCL-2, CCL-4, CCL-5, CXCL-10, and TIMP-2 were done on 1 µg total protein of endothelial cells (EC)-derived uEV, tEV, and cEV. For data of ELISA, *p values <0.05 was considered as statistically significant. Values are given as mean ± SD of three independent biological individuals in two technical replicates (*n* = 6).

There is insufficient evidence concerning the mode of action of released EV during an inflammatory stress response. In order to obtain a complete overview on the inflammatory content of the EC-EV in this study, antibody-pair-based assays and ELISA were used to detect several EV-associated inflammatory mediators simultaneously. Therefore, we first assayed the immunomodulatory content of uEV and tEV lysate using human inflammatory arrays C1 and C2 (Figure 2A). These arrays include several inflammatory markers such as cytokines, growth factors, cellular adhesion, and inflammation-associated markers. Among 40 pro- and anti-inflammatory proteins, GM-CSF, IL-6, IL-8, ICAM-1, CXCL-10, CCL-5, TNF-α, and TNF-R were significantly higher expressed in the tEV as compared to uEV (Figure 2B). We also observed that the detected intensity for CCL-2 in the tEV was slightly higher than uEV (Figure 2B).

To further confirm the array defined markers and quantify the EV pro- and anti-inflammatory protein content, ELISA-based assays for GM-CSF, IL1-β, IL-4, IL-6, IL-6R, IL-8, IL-10, IL-13, ICAM-1, CCL-2, CCL-4, CCL-5, CXCL-10, and TIMP-2 were done. ELISA analyses confirmed the expression level of IL1-β (*p* = 0.0006), IL-6 (*p* = 2.4 E−9), IL-8 (*p* = 0.0054), IL-10 (*p* = 0.006), IL-13 (*p* = 3.5 E−06), ICAM-1 (*p* = 0.0008), CCL-2 (*p* = 3.1 E−5), CCL-5 (*p* = 0.001), and CXCL-10 (*p* = 1.1 E−5) were statistically significantly increased in the tEV as compared to uEV (Figure 2C). These data already show that EV derived from inflammation-triggered EC are highly enriched with several key pro-inflammatory mediators, chemokines whereas anti-inflammatory mediators (IL-10 and IL-13) were barely expressed in them. In order to find out the role of these inflammatory EV in the cytokine and chemokine networks during inflammatory mediated cross-talk between EC and MC as well as their functional effect on these two recipients, we further investigated the physiological impact of EV derived from TNF-α stimulated HUVEC (tEV) and non-stressed (unstimulated) cells (uEV) on two major CVD cell culture models, HUVECs (reference cell culture model for EC) and THP-1 (reference cell culture model for MC) at both protein and RNA levels and functional behavior *in vitro*. In addition, negligible amounts of cytokines and chemokines were detected in EV derived from cell-free medium treated with 10 ng/ml TNF-α as negative control (Figure 2C).

### EC-EV Alter the Inflammatory Profile of MC (THP-1) and EC (HUVEC)

**Figure 3:**
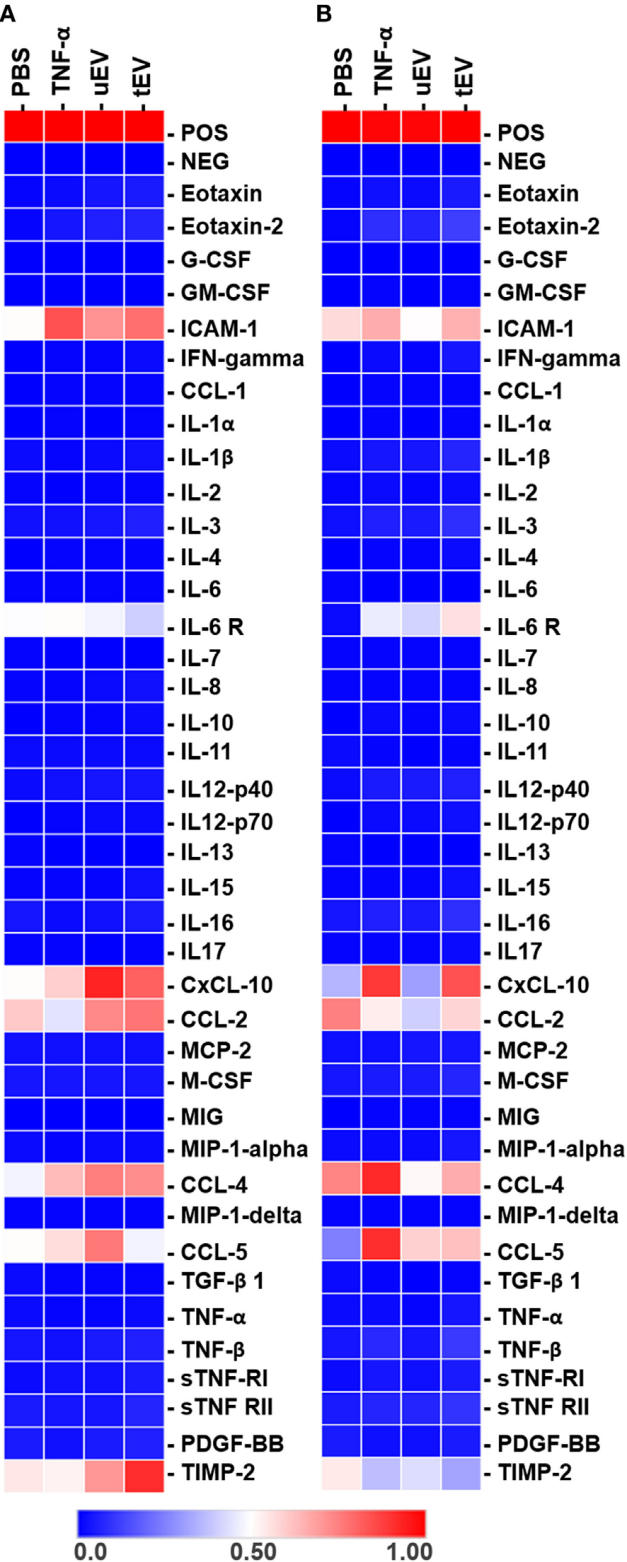
Heat map of the inflammation-related protein expression in HUVEC **(A)** and THP-1 **(B)** cells treated with PBS, TNF-α (10 ng/ml), uEV, and tEV (10 µg/ml total proteins).

To assess whether EC-EV shuttle the inflammatory-associated proteins and induce their expression in HUVEC and THP-1 at the protein level, we performed an semi-quantitatively protein array and ELISA on the cell lysate of recipient cells treated with uEV, tEV, TNF-α (positive control), and PBS (negative control) for 18 h. First, a membrane-based inflammation array C3 was used to detect the differentially expressed cytokines, growth factors, cellular adhesion, and inflammation-associated markers, concurrently (Figures 2A,B in Supplementary Material). Expression of a wide range of inflammatory markers was evident in the TNF-α and EV treated HUVEC and THP-1. Heat map analysis of differentially expressed proteins revealed that among 40 human inflammatory markers, a series of chemotactic cytokines and adhesion promoters including ICAM-1, IL-6R, CXCL-10, CCL-2, CCL-4, CCL-5, TIMP-2, and several ILs were the most highly expressed in both cell types (Figures 3A,B). Since, this method serves only a semi-quantitatively array for profiling several inflammation-associated portions, we next quantified the detected markers (ICAM-1, IL-6R, CXCL-10, CCL-2, CCL-4, CCL-5, and TIMP-2) and ILs (IL1-β, IL-4, IL-6, IL-8, IL-10, and IL-13) using ELISA.

**Figure 4:**
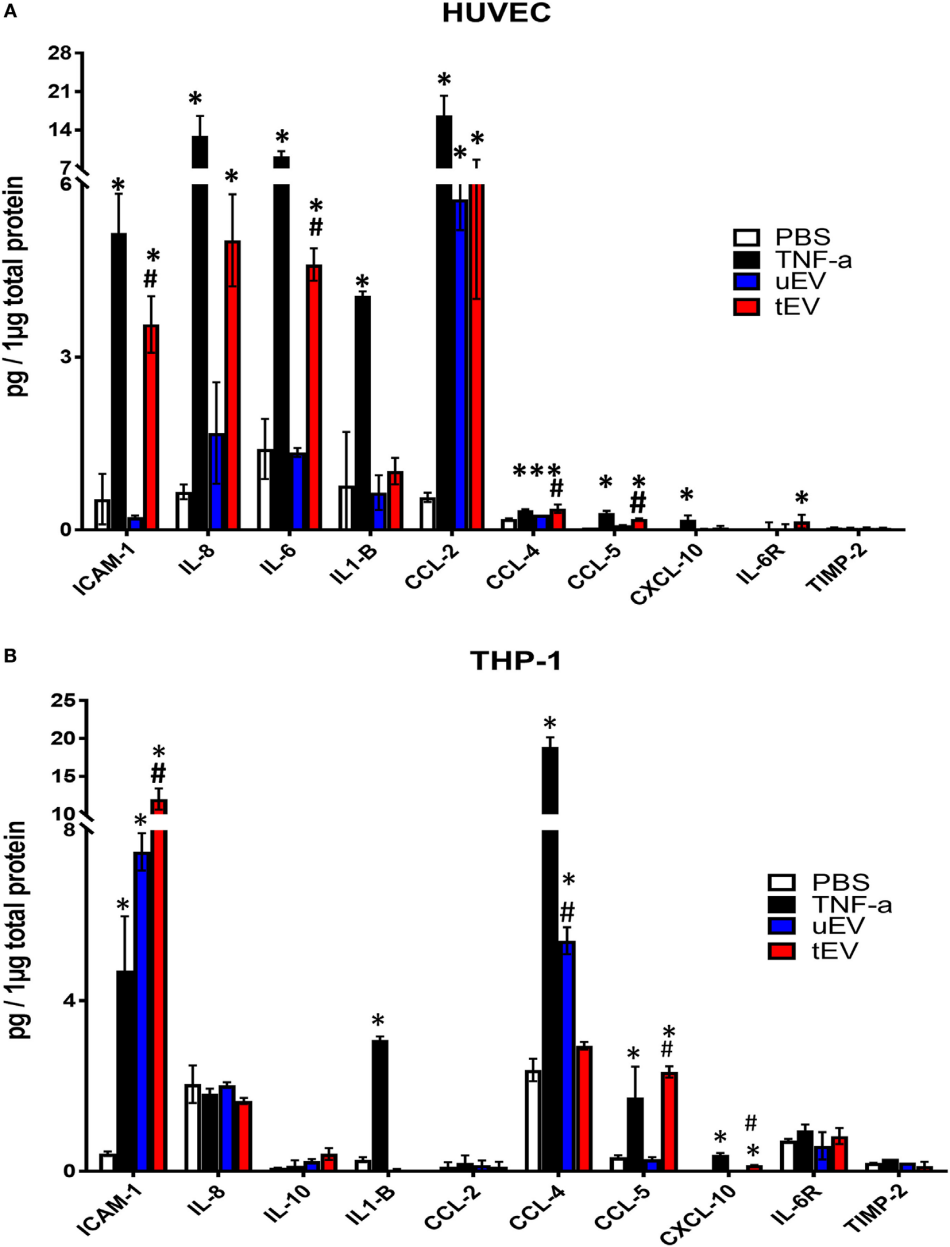
ELISA analysis of inflammatory markers including interleukins (IL1-β, IL-6, IL-6R, IL-8, IL-10, and IL-13), cellular adhesion and inflammation-associated marker [intercellular adhesion molecule (ICAM-1)] and chemokines (CCL-2, CCL-4, CCL-5, and CXCL-10) and TIMP-2 in cells extraction of HUVEC **(A)** and THP-1 **(B)** treated with PBS, TNF-α (10 ng/ml), uEV (10 µg/ml total proteins), and tEV (10 µg/ml total proteins). Values are given as mean ± SD of three independent biological individuals in two technical replicates (*n* = 6). *p*-Values were calculated by one-way analysis of variance with a multiple comparisons test (Tukey’s multiple comparison test) and *p < 0.05 (PBS vs treatments) and ^#^*p* < 0.05 (uEV vs tEV) were considered as statistically significant.

Indeed, ELISA data confirmed that a pro-inflammatory state was happened in the tEV recipient HUVEC cells by the upregulation of adhesion molecule expression, particularly ICAM-1 (ninefold, *p* = 0.0024) compared to PBS-treated HUVEC (Figure 4A). In addition to the upregulation of adhesion marker, production of pro-inflammatory cytokines and chemokines, including IL-6 (1.5-fold, *p* = 0.016), IL-8 (7-fold, *p* = 0.039), CCL-2 (11-fold, *p* = 0.007), CCL-4 (2-fold, *p* < 0.0001), CCL-5 (4-fold, *p* = 0.0097), and IL6-R (2-fold, *p* = 0.0313) markedly increased in HUVEC upon exposure to tEV in comparison with PBS-treated cell (Figure 4A). In the case of uEV-treated HUVEC, the expression of only two chemotactic chemokines, CCL-2 (10-fold, *p* = 0.016) and CCL-4 (2-fold, *p* = 0.003), was significantly increased, suggesting that transferring the immunomodulators is not limited to EV derived from triggered cells. In the case of THP-1, cells treated with EC-EV (both uEV and tEV) were significantly expressed ICAM-1 (18-fold, *p* = 0.0058 and 27-fold *p* < 0.0001, respectively), as candidate of pro-inflammatory markers. While the expression of other pro-inflammatory markers including IL-8 (*p* = 0.43) and CCL-2 (*p* = 0.99) was not significantly altered when tEV were added to THP-1 cells (Figure 4B) compared to PBS-treated cells, a marked increase in other chemotactic chemokine such as CCL-5 (7-fold, *p* = 0.0046) and CXCL-10 (12-fold, *p* = 0.0002) was observed. Addition of uEV to THP-1 were only significantly increased the production of CCL-4 (2.2-fold, *p* = 0.032) compared to PBS-treated cells (Figure 4B). As shown in Figure 4A, the expression of ICAM-1, IL-8, IL-6, IL1-β, CCL-2, CCL-4, CCL-5, and CXCL-10 markers were significantly increased in TNF-α-treated HUVEC (positive control) compared to PBS-treated cells. In THP-1, there were significant increase in the expression of ICAM-1, IL1-β, CCL-4, CCL-5, and CXCL-10 (Figure 4B) in TNF-α-treated cells compared to PBS (*p* values are presented in Table S1 in Supplementary Material). Results at the protein levels (Figures 4A,B) revealed that a pro-inflammatory behavior in HUVEC and a mix of pro- and anti-inflammatory phenotypes in THP-1 was promoted after hosting EV.

Collectively, these results suggest that EV content may selectively transfer inflammatory markers to recipients and altered their cellular profiles differently. In particular, they promoted a pro-inflammatory behavior in HUVEC, whereas they re-programmed THP-1 toward a mixed of pro- and anti-inflammatory phenotype as indicated by elevated expression of ICAM-1, CCL-4, CCL-5, and CXCL-10.

### EC-EV Increase the Expression of Adhesion Molecule (ICAM-1) in MC (THP-1) and EC (HUVEC)

**Figure 5:**
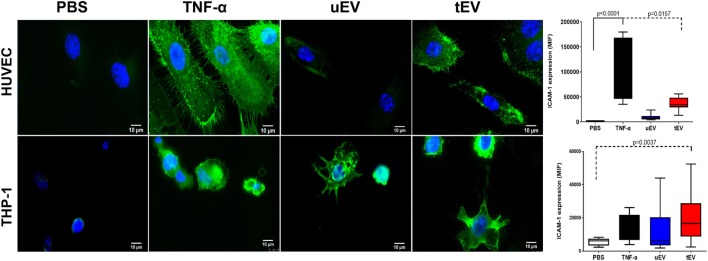
Immunofluorescence visualization of intercellular adhesion molecule (ICAM)-1 expression in HUVEC and THP-1 treated with PBS, TNFα, uEV, and tEV. Quantification of protein expression was done based on the mean of intensity of three independent samples for each stimuli. Nuclei were stained with diamidino-2-phenylindole. Values are given as mean ± SD of three independent biological individuals in three technical replicates (*n* = 9). *p*-Values were calculated by one-way analysis of variance and *p* values <0.05 was considered as statistically significant.

Intercellular adhesion molecule-1 expression is one the key candidate for inflammation-associated disorders. In our protein studies, we discovered the expression of this marker was significantly induced in HUVEC and THP-1 treated with EC-EV. Therefore, to understand if EC-EV can actively induce inflammation in EC and MC, the induction of ICAM-1 as a key candidate of inflammation was immunofluorescently visualized and quantified (Figure 5). In the line with ELISA results, expression of ICAM-1 in HUVEC after TNF-α and tEV exposure was significantly enhanced (*p* < 0.0001 and *p* = 0.0157, respectively) (Figure 5). A low level of ICAM-1 was expressed in PBS treatment HUVEC. Upon stimulation with tEV in THP-1, ICAM-1 expression was increased (*p* = 0.0037) whereas only a modest enhancement (*p* = 0.17) was detected in the uEV-treated THP-1.

### EC-EV Promote THP-1 Adhesion and Migration

**Figure 6:**
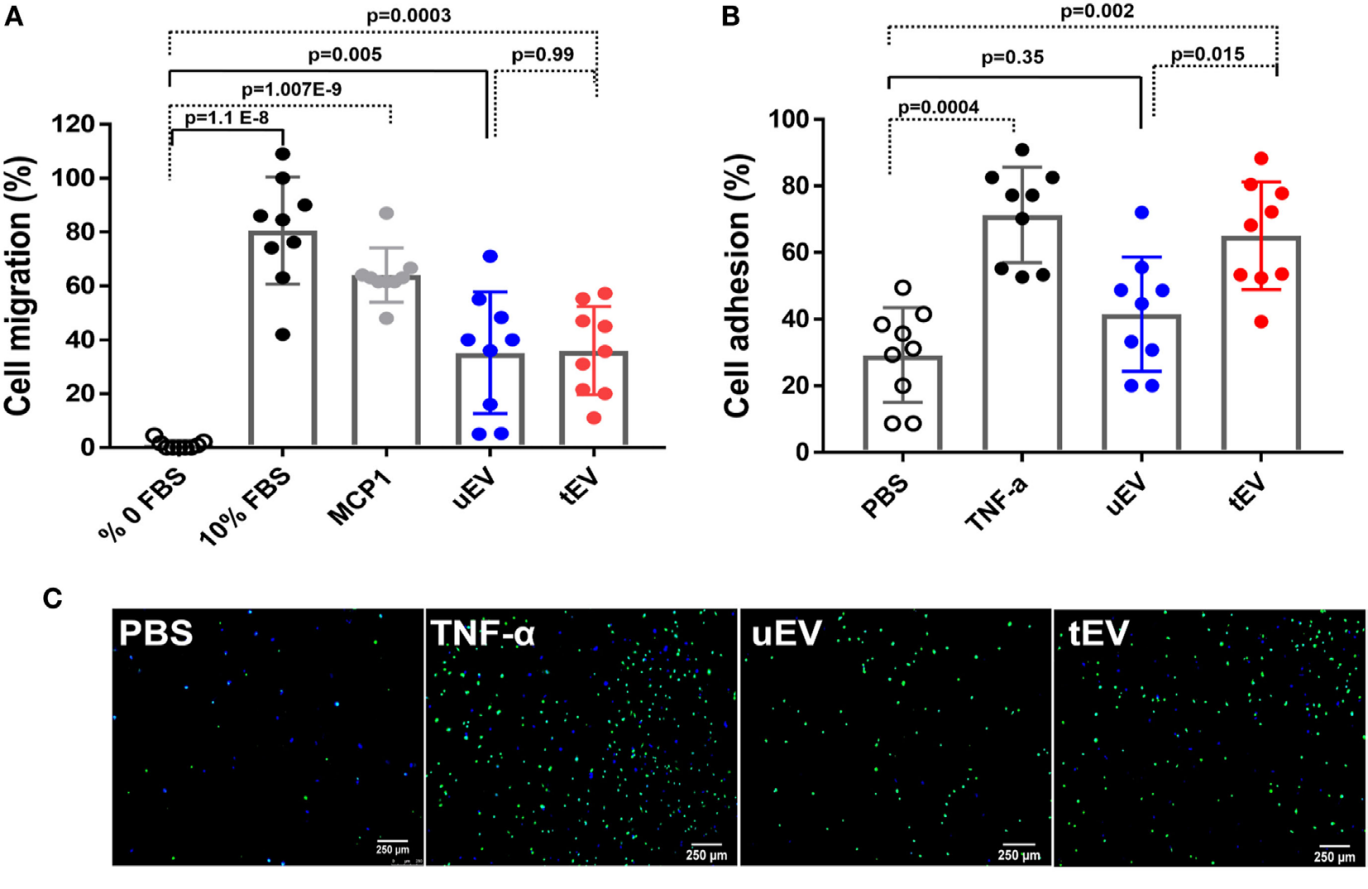
Endothelial cells (EC)-extracellular vesicles (EV) promote THP-1 adhesion and migration. **(A)** THP-1 cell migration under different treatments including 0% FBS, 10% FBS, 50 ng/ml MCP-1, 10 µg/ml total protein of uEV and tEV. **(B)** THP-1 cell adhesion to HUVEC under different treatments including PBS, 10 ng/ml TNFα, 10 µg/ml total protein of uEV and tEV. **(C)** A representative photograph of THP-1 cell adhesion to HUVEC under different treatments. Data are given as mean ± SD of three independent biological individuals in three technical replicates (*n* = 9) and one-way analysis of variance with a multiple comparisons test (Tukey’s multiple comparison test) was used to evaluate the statistical significance between different treatments.

The activation, adhesion, and transendothelial migration of MC into the intima occurs rapidly during development of atherosclerosis. As EC-EV are enriched with a cocktail of chemotaxis and migration associated factors, we further investigate whether these EV are actively involved in MC adhesion and migration. The chemotactic effect of uEVs or tEVs on the migration of THP-1 were compared with the condition without and with THP-1 migration capacities (0% FBS and 10% FBS, respectively). Our data were demonstrated a chemotactic effect of EC-EV on THP-1 by promoting their transmembrane migration in the presence of EC-EV using in an *in vitro* transwell migration assay. As shown in Figure 6A, when THP-1 was incubated with uEV and tEV, THP-1 migration enhanced by 32 ± 22.5 and 35 ± 16.7%, respectively (mean ± SD, *n* = 9) compared to 0% FBS (Figure 6A). In the response to 10% FBS and MCP-1, positive controls, THP-1 migration were increased up to 80.5 ± 20 and 64 ± 10.1%, respectively.

Also, a functional adhesion assay was performed to discover the effect of EC-EV at the crossing of inflammation and development of vascular disease by measuring the adhesion of THP-1 monocytes to HUVEC monolayer under static conditions. As shown in Figures 6B,C, pre-incubation of HUVEC with either tEV or TNF-α effectively increased the adhesion of THP-1 (*p* = 0.002 and *p* = 0.004, respectively) as compared to PBS-treated HUVECs. Exposure of HUVEC to uEV has a slight but not significant effect on THP-1 adhesion as compared to PBS-treated cells (*p* = 0.35) but there was a statistically significant difference between uEV and tEV (*p* = 0.015) treated cells. These observations confirmed the role of EV in the transferring biologically active inflammatory modulators across cells which lead to amplifying the inflammatory response by EC activation and MC adhesion and migration.

## Discussion

### The Combined of Small and Large EV Together Are Associated With Inflammation

Prior studies have revealed the role of small EV (exosomes) and large EV (microvesicles) in the progression of the inflammatory associated diseases individually (19, 20). Moreover, it has been observed that during the development of inflammation-associated disorders, a multitude of diseased and healthy cells are constitutively releasing a heterogeneous mixture of both small and large EV into the body fluids (8). These combined EV are actively contributing to natural intercellular communication *via* transporting several bioactive molecules such as peptides, proteins, lipids, mRNAs, and miRNAs (19). Yet, the content and functionality of the combined fraction of both small and large EV are still missing. To our knowledge, this is the first study to investigate the immunomodulatory content of the combined small and large EV derived from inflamed vascular cells and to discover their effect on the cellular fitness and function of recipients. In order to isolate a combined fraction of both small and large EV, the collected supernatant was first centrifuged at 300 and 2,000 *g* to eliminate cell debris and apoptotic bodies, respectively (14). Pelleting of large and small EV together were then happened at 110,000 *g*. Principally, in the differential centrifugation method, the most commonly used protocol for EV isolation, small and large EV are separated at different g-forces and k-factors. As fractioning of large EV (microvesicles) and small EV (exosome) from different cell types could be done at g-forces of 10,000–20,000 and >100,000 *g*, respectively (14). Therefore, the co-pelleting of small and large EV was done by skipping the 10,000–20,000 *g* centrifugation step (Figure 1A; Figure S1 in Supplementary Material).

### EC-EV Contain Several Inflammatory Mediators

Several studies have demonstrated that the initiation and progression of inflammation-associated disorders such as atherosclerosis and CVD are governed by interactions between EC and MC *via* multiple inflammatory mediators, the best recognized of which are cell adhesion molecules (e.g., ICAM-1), chemoattractants (e.g., CCL-2, CCL-4, and CCL-5), growth factors (e.g., GM-CSF), and cytokines (e.g., IL-6, IL-8) (2, 20, 21). Although, it is well known that chemokines and cytokines are effectively involved in a complex inflammatory interaction between EC and circulating immune cells, little is known about the EC-EV immunomodulatory content and their role in the chemokine network between the two key drivers (EC and MC) after an inflammatory stress response.

In our previous study, we already demonstrated that an elevated level of ICAM-1(+) small EV were released from inflammation-triggered EC (16). To our knowledge, this study presents the first complete overview of the common immunomodulatory content of the combined fraction of both small and large EV released from inflammatory-triggered EC. Our data suggest that beyond the higher expression of adhesion markers (ICAM-1) in EV derived from inflammation-triggered vascular EC, these EV contain several pro-inflammatory mediators including chemotactic mediators such as IL-6, IL-8, CXCL-10, monocyte chemoattractant protein-1 (CCL-2), macrophage inflammatory protein (CCL-4 and CCL-5) together with key anti-inflammatory mediators (IL-10 and IL-13). These EV enriched with a cocktail of inflammatory agents may contribute in the earliest phase of atherosclerosis and CVD which is initiating by endothelial dysfunction, recruiting monocytes/macrophages toward EC and then rolling and transendothelial migration of MC into the intima.

### EC-EV Mediate Inflammatory Responses in EC and MC

Previous studies have shown that RNA content of EV-EC are mainly playing a central role in the educating recipient cells toward inflammatory gene activation or suppression responses (22). However, we show that EC-EV harbor a wide range of inflammatory proteins, suggesting that EV-associated proteins could attribute to the functional activity of recipient cells. Several studies have already demonstrated that EV may transfer inflammation-associated protein (e.g., ICAM-1) into their target cells (23, 24). Here, comparing whole protein profiles of cell lysate with the EV content (Figures 2 and 3) highlight that EV may be able to selectively transfer the specific inflammatory associated mediators to target cells (e.g., CCL-5 and CXCL-10 to THP-1 and ICAM-1, IL-6 and IL-8 to HUVEC) thereby modulate cells toward either pro-inflammatory (HUVEC) or pro/anti-inflammatory (THP-1) statues. Moreover, the elevated expression of ICAM-1, IL-6, and IL-8 in tEV-treated HUVEC, suggest that EV may translocate these pro-inflammatory mediators and promote vascular endothelial inflammation. In fact, ICAM-1 together with IL-6, IL8 play an important role in the progression of atherosclerosis through triggering the transendothelial migration of immune cells to the site of inflammation and the activation of pro-inflammatory cascades in target cells (5, 7, 21). We also provide evidence that chemokine-enriched EV (tEV) can modulate the expression of anti-inflammatory markers including CCL-5 and CXCL-10 in THP-1. Overall, a broad range of pro-inflammatory proteins in HUVEC and pro/anti-inflammatory proteins in THP-1 were significantly induced by the bulk of both uEV and tEV compared to the control. It is likely that specific modulators contained in EV may play these extensive inflammatory effects and regulate the expression of a large number of inflammatory-associated genes. The changes in the phenotype and behavior of recipient cells in this time frame of treatment (an overnight incubation) can be associated with either the transfer of the EV cargo into cells or *de novo* synthesis of inflammatory markers induced by the EV cargo or can be due to a mixture of both pathways. While the impact of EC-EV on the two target cells was investigated in this study, the actual mechanistic pathway of EV involved in these effects as well as their uptake/transfer pathway into recipients are still unclear and needs to be further investigated. Yet, another key mediator for the inflammatory effect of EV would be their RNA cargo. Further investigation is therefore required to detect the RNAs-associated inflammation in the EV derived from inflammatory-triggered EC, profile changes at the transcriptional level and to discover their functional contribution in MC adhesion and mobilization. In this work, both tEV and uEV were first isolated from the same number of parent cells. The total protein concentration of tEV was higher than uEV from the same number of parent cells. In addition, as presented in the Figure S1 in Supplementary Material, higher concentrations of particle number/ml EV was detected in TNF-α stimulated HUVEC (tEV) when compared to non-stressed (unstimulated) cells (uEV). In the next step, to understand the effect of EV fractions we normalized EV samples for functional assays where we considered both criteria (particle number and protein concentration). As recommended by Tkach et al. 2018, the combined quantification of total protein and particle number is the best way to quantify materials present in an EV preparation (25). Adjusting both uEV and tEV to 10 µg/ml the total protein was fairly balanced to the same number of EV (e.g., ~1E9). In our opinion, target cells were undergoing the same uEV and tEV treatments.

### EC-EV Are Related to Migration and Adhesion of MC

The majority of studies has been focused on the functional properties of EV derived from MC at the crossing of inflammation and development of vascular disease (24, 26). EC-EV are most likely to be an important coordinator in the cardiovascular homeostasis, maintaining cardiac functions and development of CVD due to the position of their parental origin, EC, at the interface of vascular cells and immune cells. Here, our study shows that EC-EV are not only mediated the THP-1 adhesion into HUVEC but are also capable of promoting their transmembrane migration *in vitro*. Actually, docking of EV proteins into HUVEC and THP-1 induces the expression of key chemotactic mediators including IL-6, IL-8, CXCL-10, monocyte chemoattractant protein-1 (CCL-2), and macrophage inflammatory protein (CCL-4 and CCL-5), leading to increased THP-1 adhesion and mobilization. At the functional level, our results also support the idea that EC-EV are involved in the chemokine networks between EC and MC at sites of inflammation, in particular, promoting the MC adhesion into EC and recruiting them to inflamed sites. Taken together, our study revealed that EC-EV are actively associated with the vascular endothelial inflammation, MC-associated inflammatory response and MC adhesion and migration. In addition, our results extended the previous findings regarding EV mediated an inflammatory cross-talk between EC and their neighboring EC and circulating MC and we for the first time report that this intercellular communication seems most likely occurring *via* EV-mediated transferring of inflammatory chemokines and cytokines to their local and distant recipients. It should be noted that our conclusion is driven from *in vitro* studies at the protein and functional levels. *In vivo* (animal) and *ex vivo* experiments are planned to explore further the involvement of EV in the communication networks at RNA, protein and functional levels.

## Author Contributions

BH, LM, NA, and DM designed the experiments. BH conducted most of the experimental work, data analysis, and interpretation, and drafted and edited the manuscript. SK conducted parts of the experimental work and revised the manuscript. LM, NA, and DM conducted several critical revisions.

## Conflict of Interest Statement

The authors declare that the research was conducted in the absence of any commercial or financial relationships that could be construed as a potential conflict of interest.
