# Supplementary material for: Extracellular Vesicles Work as a Functional Inflammatory Mediator Between Vascular Endothelial Cells and Immune Cells
Source: Front Immunol. 2018 Aug 6;9:1789. doi: 10.3389/fimmu.2018.01789 (PMC6091278; doi:10.3389/fimmu.2018.01789)
Supplement: Supplementary file 1 [file Data_Sheet_1.docx]

Supplementary Material

**Extracellular vesicles work as a functional inflammatory mediator between vascular endothelial cells and immune cells**

**Baharak Hosseinkhani^1^*****, Sören Kuypers^1^, Nynke M.S. van den Akker^2^, Daniel G.M. Molin^2^, Luc Michiels^1^**

^1^ Biomedical Research Institute, Department of Medicine and Life Sciences, Hasselt University, Hasselt, Belgium

^2^ Cardiovascular Research Institute Maastricht (CARIM), Department of Physiology, Maastricht University, Maastricht, The Netherlands

*** Correspondence:**Dr. Baharak Hosseinkhani
Baharak.hosseinkhani@uhasselt.be

# Supplementary Figures


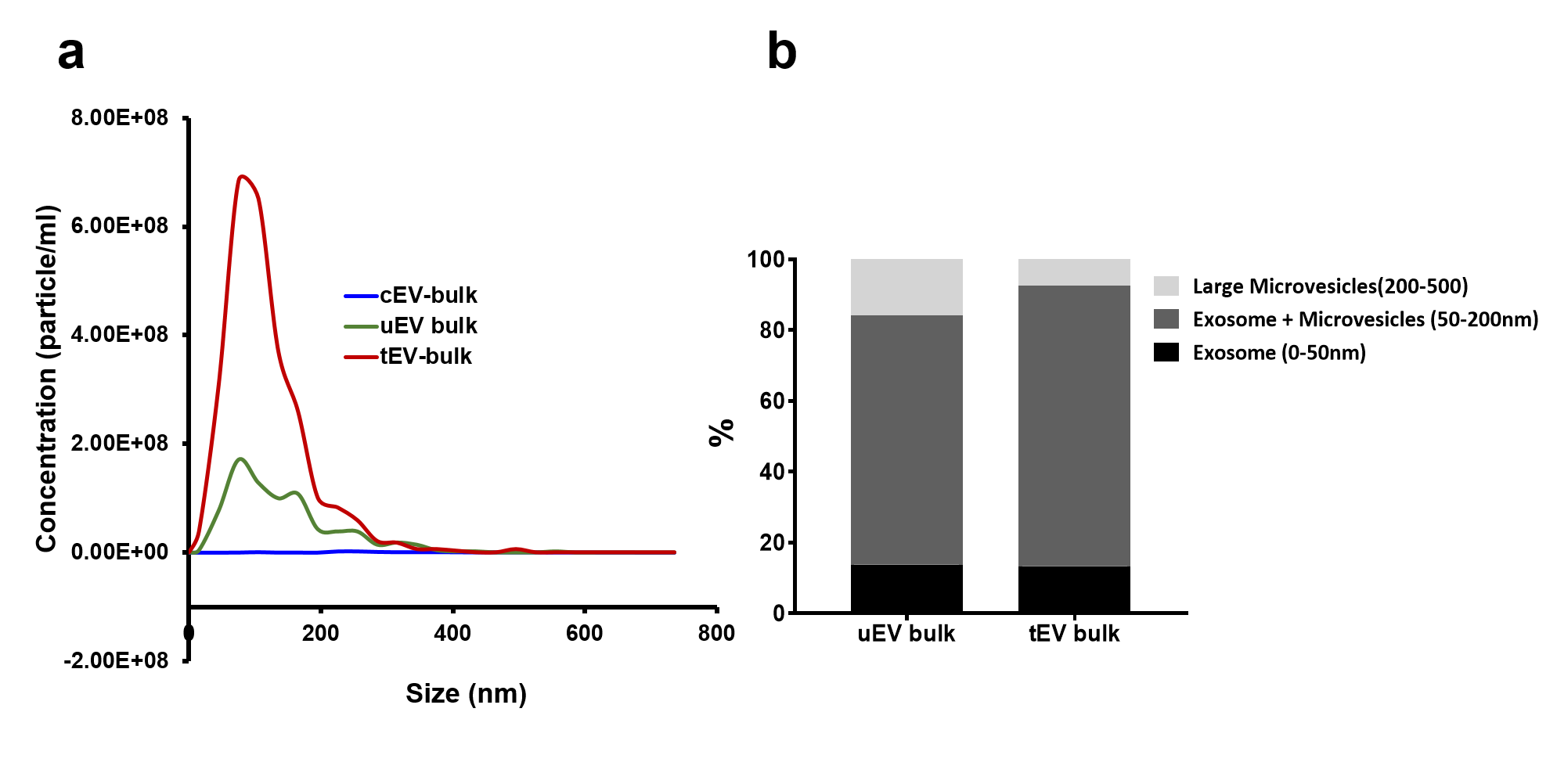


**Supplementary Figure 1.** (a) Size distribution profile and concentration of EV derived from culture supernatant of un-stimulated (uEV), TNF-α stimulated (tEV) and cell free medium (cEV). (b) The percentage of isolated bulk uEV and from tEV in the size range of exosomes (0-50 nm), exosomes + microvesicles (50-200 nm) and large microvesicles (200-500 nm).


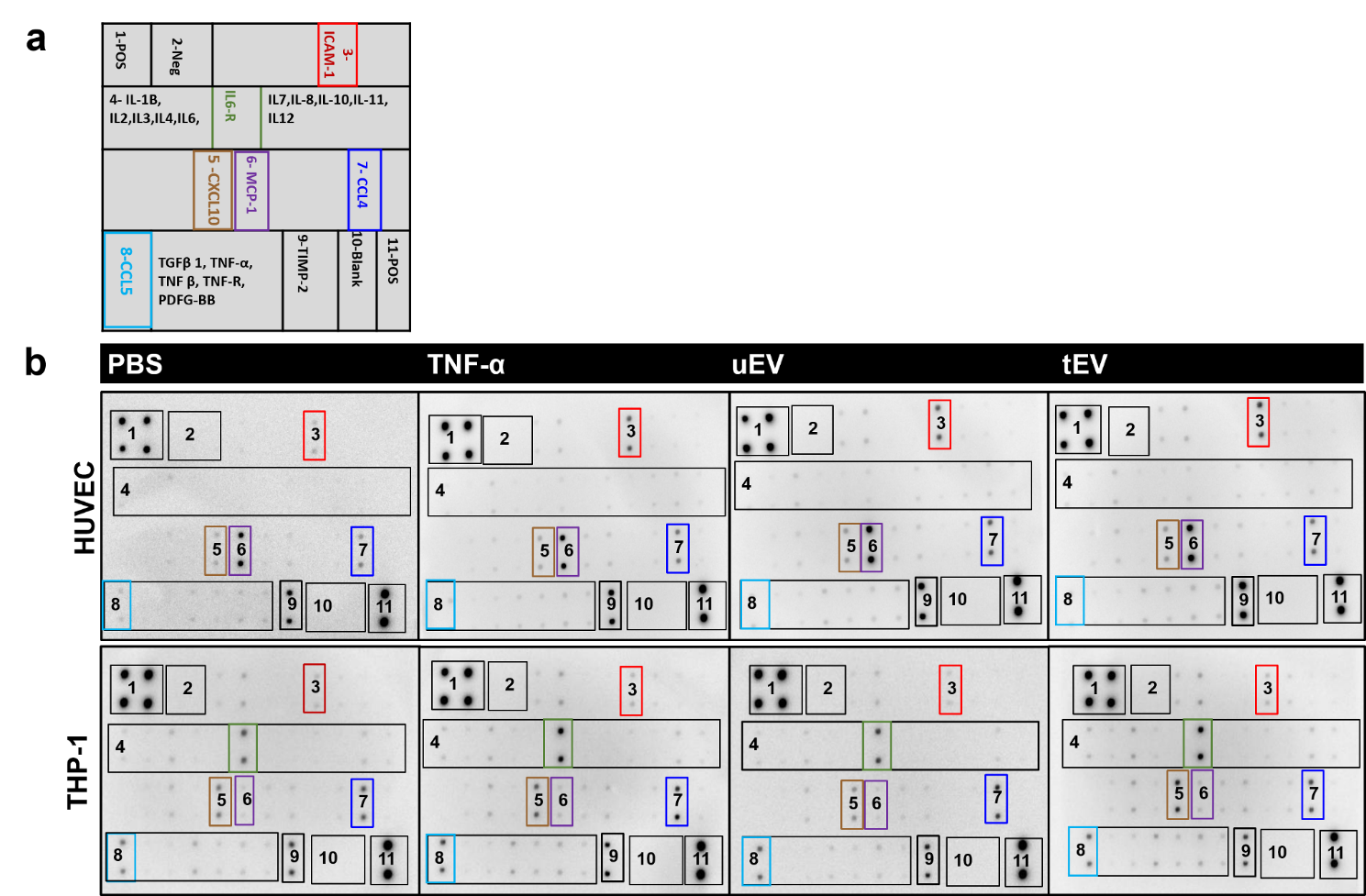


**Supplementary Figure 2:** (a) Representative image of membrane based inflammation arrays C3. (b) Protein expression profile of cell lysates of HUVEC and THP-1 treated with PBS, TNF-α, uEV and tEV.

**Supplementary table1:**

The p value between different treatments calculated by one-way analysis of variance (ANOVA) with a multiple comparisons test (Tukey’s multiple comparison test) using the statistical packages GraphPad Prism 7.04 software (GraphPad Software, Inc., La Jolla, California, USA). Values of *p< 0.05 were considered as statistically significant for PBS *vs* other treatments and # p< 0.05 were considered as statistically significant for uEV vs. tEV (green boxes) for HUVEC and THP-1.

| HUVEC ANOVA Tukey test | Adjusted P Value | |  |  |  |  |  |  |  |  |
| --- | --- | --- | --- | --- | --- | --- | --- | --- | --- | --- |
|  | ICAM-1 | IL-8 | IL6 | IL1-B | CCL-2 | CCL4 | CCL-5 | CXCL-10 | IL-6R | TIMP-2 |
| PBS vs. TNF | 0.0003 | <0.0001 | 0.0005 | 0.0024 | <0.0001 | 0.0002 | 0.0015 | 0.0004 | 0.2789 | 0.8381 |
| PBS vs. uEV | 0.9873 | 0.534 | 0.9994 | 0.9943 | 0.0163 | 0.0034 | 0.4177 | 0.9984 | 0.2502 | 0.6705 |
| PBS vs. tEV | 0.0024 | 0.0391 | 0.0159 | 0.966 | 0.0077 | <0.0001 | 0.0097 | 0.5477 | 0.0313 | 0.8712 |
| uEV vs. tEV | 0.0018 | 0.351 | 0.0149 | 0.9157 | 0.9675 | 0.0002 | 0.0287 | 0.6418 | 0.5797 | 0.9796 |
|  |  |  |  |  |  |  |  |  |  |  |
| THP-1 ANOVA Tukey test | Adjusted P Value | |  |  |  |  |  |  |  |  |
|  | ICAM-1 | IL-8 | IL-10 | IL1-B | CCL-2 | CCL4 | CCL-5 | CXCL-10 | IL-6R | TIMP-2 |
| PBS vs. TNF | 0.0058 | 0.7723 | 0.2515 | <0.0001 | 0.7384 | <0.0001 | 0.0213 | <0.0001 | 0.3699 | 0.4668 |
| PBS vs. uEV | 0.0006 | 0.9998 | 0.6298 | 0.1161 | 0.9833 | 0.0327 | 0.9986 | 0.9998 | 0.8313 | 0.9994 |
| PBS vs. tEV | >0.0001 | 0.4332 | 0.1652 | 0.1112 | 0.9997 | 0.822 | 0.0046 | 0.0002 | 0.8712 | 0.8597 |
| uEV vs. tEV | 0.0079 | 0.4677 | 0.5415 | 0.2212 | 0.9924 | 0.0639 | 0.0062 | 0.0005 | 0.4117 | 0.803 |
